# Patient Safety ‘Through Undergraduate Medical Students' Eyes’: A Mixed‐Methods Survey

**DOI:** 10.1111/tct.70332

**Published:** 2025-12-18

**Authors:** Anna Pappa, Athanasios Xymitidis, Thomas Strymponis, Paraskevi Panagopoulou, Válter R. Fonseca, Henrique Martins, João Breda, Zoi Tsimtsiou

**Affiliations:** ^1^ Department of Hygiene, Social‐Preventive Medicine and Medical Statistics, School of Medicine Aristotle University of Thessaloniki Thessaloniki Greece; ^2^ 4^th^ Pediatric Clinic, School of Medicine Aristotle University of Thessaloniki Thessaloniki Greece; ^3^ WHO Athens Quality of Care and Patient Safety Office World Health Organization Regional Office for Europe Athens Greece

## Abstract

**Background:**

Evidence is limited internationally regarding medical students' perceptions and training needs in patient safety, following the WHO Global Patient Safety Action Plan 2021–2030, the COVID‐19 pandemic, and the subsequent acceleration in digitalization. To address this gap, this study explores undergraduate medical students' knowledge of patient safety, attitudes regarding safety culture, experiences with errors, and their perspectives on improving patient safety through training and digitalization.

**Methods:**

A mixed‐method design was employed, consisting of an online survey with open‐ended questions administered at a medical school in Greece. All registered undergraduate students were invited to complete a pretested 46‐item study tool, including demographics, the Greek version of the scale ‘What is patient safety?’ and their personal experiences, views on digitalization and educational needs.

**Results:**

A total of 638 students from all 6 years of study participated (58% female; 54.2% preclinical students). Findings revealed poor knowledge regarding error management (mean 2.4/5) and the reporting process (mean 1.8/5). Fear of blame culture was reported by 51.2%. Digitalization was expected to advance patient safety in multiple ways. Students advocated strengthening patient safety training (92.8%), emphasizing case‐based learning, integrating patient safety principles into the teaching of all clinical courses and watching their professors acting as role models.

**Conclusions:**

Medical students feel underprepared to manage patient safety issues and request more relevant knowledge and skills. These findings highlight the urgency for appropriate training reforms, tailored to the students' needs, aiming to optimally prepare them to become key players in improving patient safety in a digitally evolving healthcare system.

## 1 Introduction

In 2000, the Institute of Medicine report ‘To Err Is Human: Building a Safer Health System’ brought patient safety to the forefront. It underscored the global imperative to minimize the risk of preventable adverse events and their potential impact, establishing patient safety as a key component of healthcare quality [1]. In line with this approach, the World Health Organization (WHO) developed the ‘Global Patient Safety Action Plan 2021–2030’, setting a framework of actions to eliminate preventable harm [2].

Despite the plethora of interventions aiming to improve patient safety, adverse events continue to occur at an alarming frequency. A predominant obstacle has been the prevailing workplace culture and the organizational structure [3]. Educating and raising awareness among health professionals on safety, their commitment to intended standards and the encouragement of active participation through appropriate incentives are crucial for implementing quality improvement initiatives [4]. Previous research supports that introducing quality improvement and safety culture early during undergraduate years is essential [5] as it leads to the development of relevant attitudes and skills that empower students to manage clinical challenges and optimize patient outcomes while acting as valuable collaborators in the prevention of healthcare‐related errors [6, 7].

Considering that the higher education sector has not kept up with the requirements of clinical demands, it was imperative to integrate appropriate training into the curriculum of Health Sciences Schools. In this direction, WHO developed the Patient Safety Curriculum Guide for Medical Schools, as well as a multi‐professional edition [8, 9]. Since then, pilot educational procedures have been implemented, including core content areas such as safety concepts, root cause analysis, teamwork, communication skills and quality improvement principles, with positive feedback from trainees [10].

In many countries worldwide, including Greece, patient safety is not systematically taught, though its principles are presented in various preclinical and clinical courses in medical schools' curricula. The newly introduced National Strategy for Quality of Care and Patient Safety (2025–2030) has included the incorporation of modules in quality of care and patient safety in undergraduate curricula for all healthcare professionals among the set priorities [11]. However, evidence is limited internationally regarding the current medical students' perceptions and training needs in patient safety, adapting to global turning points such as the COVID‐19 pandemic, the subsequent acceleration in digitalization and the introduction of innovative educational approaches. Mapping students' perspectives regarding patient safety is necessary in light of the recent developments in better informing teaching strategies. To address this gap, this study aimed to explore medical students' knowledge on patient safety issues, their attitudes regarding safety culture, their experiences on errors and their perspectives on improving patient safety through training and digitalization.

*Evidence is limited internationally regarding the current medical students' perceptions and training needs in patient safety, adapting to global turning points such as the COVID‐19 pandemic*.

## 2 Materials and Methods

### 2.1 Participants and Survey Administration

A mixed‐method design was employed, incorporating an online survey with open‐ended questions administered to all actively enrolled undergraduate students across all years of study in the School of Medicine of Aristotle University of Thessaloniki. Undergraduate medical education in Greece follows a 6‐year curriculum, with the first 3 years dedicated mainly to preclinical studies and the following 3 years to clinical rotations. During the winter semester of 2023–2024, students were invited to participate anonymously and voluntarily via two emails, distributed 2 weeks apart by the school's administration, including an informational note and a link to the questionnaire. Informed consent was obtained prior to questionnaire completion. To increase participation, printed QR codes linking to the questionnaire were provided to students during mandatory courses. Inclusion criteria included being actively enrolled at the School and adequate understanding of Greek. Therefore, Erasmus students were excluded.

*A mixed‐method design was employed, incorporating an online survey with open‐ended questions administered to all actively enrolled undergraduate students across all years of study*.

### 2.2 Survey Development

The survey was developed after a thorough literature search and consultation with international experts from the WHO Athens Quality of Care and Patient Safety Office. It is comprised of three parts (Data S1). The first part consisted of the 30‐item Greek validated version of the WHO questionnaire ‘What is patient safety?’, as well as the three additional items of the 33‐item original scale that were not included in its validated Greek version due to the results of confirmatory factor analysis [12, 13]. The Greek version consists of the following subscales: Error and patient safety (7 items), Health system safety and individual action (10 items), Personal attitudes to patient safety (4 items), Error and blame attribution (3 items), and Patient safety culture at the workplace (6 items). A five‐point Likert scale was used, where a higher score indicates a greater level of knowledge (Subscale 1) and more positive attitudes towards the safety culture (Subscales 2–5). This instrument was selected over other available tools as it was created for the predelivery and postdelivery evaluation of the WHO Patient Safety Curriculum Guide [8].

*The survey was developed after a thorough literature search and consultation with international experts from the WHO Athens Quality of Care and Patient Safety Office*.

The second part assessed students' views on the Greece's national patient safety plan, digitalization as a means of improving patient safety, microbial resistance, the financial burden due to errors and induced harm, experiences of errors and perceptions of their patient safety training. A 10‐point Likert scale ranging from 0 (*no degree*) to 10 (*maximum degree*) and dichotomous questions (yes/no), as well as open‐ended questions, were used. The section concluded with an open‐ended question on students' training needs and expectations.

Demographic data were gathered in the third section (age and gender) as well as their year of study. The study questionnaire required approximately 10 min to complete. It was pretested in a convenience sample of 10 undergraduate students, comprising male and female students from all years of study (these data were not included in the analysis). Participants' feedback indicated good comprehension with no difficulties in interpreting specific concepts and no need for modifications.

### 2.3 Data Analysis

Data analysis was conducted using Jamovi software (Version 2.3.2021). Normality of quantitative variables was assessed using the Shapiro–Wilk test. Variables with normal distribution were described with mean (M) and standard deviation (SD) and were analyzed with Student's *t* test and Welch's *t* test. Otherwise, the median (Mdn) and interquartile range (IQR) were reported, and the Mann–Whitney *U* test was employed. For dichotomous variables, the chi‐square (*χ* ^2^) test of independence was conducted. In all cases, statistical significance was set at a *p* value < 0.05.

Qualitative data from open‐ended responses were analyzed by three independent researchers using thematic content analysis. The coding was compared and discussed until reaching consensus by the research team.

### 2.4 Ethical Considerations

The study protocol was approved by the Bioethics Committee of the School of Medicine of Aristotle University of Thessaloniki (protocol number 57/10.11.2023). Participation was anonymous and voluntary. No incentives were provided.

## 3 Results

### 3.1 Demographics of Participants

A total of 638 undergraduate medical students participated (response rate 32%), out of which 54.2% (*n* = 346) were in the preclinical years. Table 1 presents the detailed characteristics of the participants.

**TABLE 1:** Participants' characteristics (*N* = 638).

Characteristic	*N* (%)/mean (SD)
Age (years)	21.8 (3.5)
Gender	
Female	370 (58%)
Male	263 (41.2%)
Nonbinary	2 (0.3%)
Transgender	1 (0.2%)
Agender	1 (0.2%)
They–them	1 (0.2%)
Year of study	
1st year	65 (10.2%)
2nd year	109 (17.1%)
3rd year	172 (27%)
4th year	92 (14.4%)
5th year	174 (27.3%)
6th year	26 (4%)

### 3.2 Knowledge and Attitudes Towards Patient Safety

Students exhibited positive perceptions of patient safety issues, as the subscale ‘Personal attitudes to patient safety’ received the highest score (4.2/5), whereas ‘Error and patient safety’, which evaluates the level of relative knowledge, showed the lowest score (2.5/5). Table 2 presents mean scores and standard deviations in participants' responses to the 33 items of the original WHO ‘What is patient safety?’ questionnaire, as well as the five subscales of its validated Greek version with a comparison between the preclinical and clinical years of study. Participants self‐assessed their knowledge as limited regarding the types of human errors, contributing factors, speaking up about errors, management methods and the official reporting process, with the last question recording the lowest percentage of positive responses.

**TABLE 2:** Mean scores and standard deviations in participants' responses to all the 33‐items of the original WHO ‘What is patient safety?’ questionnaire, as well as of the five subscales of its validated Greek version with comparison between the preclinical and clinical years of study.

Subscales (Cronbach's *α*)/items	Mean (SD)	Preclinical years mean (SD)	Clinical years mean (SD)	*p*
Error and patient safety (Cronbach's *α*: 0.84)	2.5 (0.5)	2.5 (0.8)	2.6 (0.7)	0.030
Level of knowledge regarding				
Different types of human error?	2.7 (1.0)	2.6 (1.0)	2.8 (0.9)	0.008
Factors contributing to human error?	3.0 (1.0)	2.9 (1.0)	3.1 (0.9)	< 0.001
Factors influencing patient safety?	3.1 (1.0)	3.0 (1.0)	3.2 (0.9)	0.064
Ways of speaking up about error?	2.4 (1.0)	2.4 (1.0)	2.4 (1.0)	0.944
What should happen if an error is made?	2.4 (1.1)	2.4 (1.0)	2.4 (1.0)	0.072
How to report an error?	1.8 (1.0)	1.8 (1.1)	1.9 (1.0)	0.188
The role of healthcare organizations (e.g., hospitals, general practitioners) in error reporting?	2.2 (1.1)	2.2 (1.2)	2.1 (1.0)	0.633
Health system safety and individual action (Cronbach's *α*: 0.68)	2.9 (0.4)	2.9 (0.4)	2.9 (0.5)	0.083
Most healthcare workers make errors.^†^	2.6 (0.8)	2.6 (0.8)	2.5 (0.9)	0.702
In my country there is a safe system of healthcare for patients.	2.8 (0.8)	2.8 (0.8)	2.7 (0.9)	0.380
Medical error is very common.^†^	2.7 (0.8)	2.7 (0.8)	2.7 (0.8)	0.338
It is very unusual for patients to be given the wrong drug.	2.8 (0.9)	2.7 (0.8)	2.8 (0.9)	0.213
Healthcare staff receive training in patient safety.	3.3 (0.9)	3.4 (0.9)	3.1 (1.0)	< 0.001
Telling others about an error I made would be easy.	2.9 (0.9)	2.9 (0.9)	2.9 (1.0)	0.547
I am confident about speaking to someone who is showing a lack of concern for a patient's safety.	3.3 (1.0)	3.4 (1.0)	3.3 (1.0)	0.316
I know how to talk to people who have made an error.	2.8 (0.9)	2.9 (0.9)	2.8 (0.9)	0.718
I am always able to ensure that patient safety is not compromised.	2.5 (0.8)	2.6 (0.8)	2.4 (0.9)	0.049
I am able to talk about my own errors.	3.5 (0.8)	3.4 (0.8)	3.5 (0.8)	0.057
Personal attitudes to patient safety (Cronbach's *α*: 0.76)	4.2 (0.5)	4.2 (0.6)	4.3 (0.5)	0.518
By concentrating on the causes of incidents I can contribute to patient safety.	3.9 (0.7)	3.9 (0.7)	4.0 (0.7)	0.010
If I keep learning from my mistakes, I can prevent incidents.	4.2 (0.7)	4.2 (0.7)	4.2 (0.7)	0.643
Acknowledging and dealing with my errors will be an important part of my job.	4.3 (0.7)	4.4 (0.7)	4.3 (0.6)	0.377
It is important for me to learn how best to acknowledge and deal with my errors by the end of medical school.	4.5 (0.7)	4.4 (0.7)	4.5 (0.6)	0.672
Error and blame attribution (Cronbach's *α*: 0.80)	2.6 (0.9)	2.6 (0.9)	2.7 (1.0)	0.16
The nurses will not criticize me for making mistakes.	2.7 (1.0)	2.6 (1.0)	2.7 (1.0)	0.268
The doctors will not criticize me for making mistakes.	2.6 (1.0)	2.5 (1.0)	2.6 (1.0)	0.163
Managers in the healthcare system will be more interested in meeting performance targets than in patient safety.	2.5 (0.9)	2.0 (0.0)	2.56 (0.9)	0.378
Patient safety culture at the workplace (Cronbach's *α*: 0.78)	3.5 (0.6)	3.5 (0.6)	3.5 (0.6)	0.355
The nurses will be committed to identifying and addressing patient safety risks.	3.4 (0.9)	3.4 (0.9)	3.5 (0.9)	0.755
The doctors will be committed to identifying and addressing patient safety risks.	3.7 (0.8)	3.7 (0.8)	3.7 (0.8)	0.243
Managers in the healthcare system will make it easy to report errors.	3.4 (1.0)	3.4 (0.9)	3.3 (1.0)	0.548
Managers in the healthcare system will expect us to focus on patient safety.	3.8 (0.8)	3.8 (0.8)	3.8 (0.8)	0.806
Being open and honest about the mistakes I make will be acceptable at my place of work.	3.5 (1.0)	3.5 (1.0)	3.4 (1.0)	0.125
Admitting an error I had made would lead to just and fair treatment by management.	3.4 (1.0)	3.4 (0.9)	3.2 (1.0)	0.069
Items of original scale not included in its validated Greek version				
About 1 in 10 hospital patients across the world will experience some kind of adverse event.	3.9 (0.7)	3.9 (0.8)	3.8 (0.7)	0.214
It is easier to find someone to blame rather than focus on the causes of error.	3.5 (1.1)	3.5 (1.0)	3.5 (1.1)	0.733
I believe that filling in reporting forms will help to improve patient safety.	3.7 (0.8)	3.7 (0.8)	3.8 (0.8)	0.400

*Participants self‐assessed their knowledge as limited regarding the types of human errors, contributing factors, speaking up about errors, management methods and the official reporting process, with the last question recording the lowest percentage of positive responses*.

Clinical‐year students scored statistically significantly higher overall with the greatest discrepancy concerning knowledge about the types of errors and the contributing factors. At the same time, they more readily recognized the contribution of error cause analysis to future improvements in the safety of the care provided. Conversely, first‐year students were more confident in ensuring patient safety, with the majority expecting that healthcare professionals would be trained in this field. Concerning gender differences, the only significant observations were that female students reported higher knowledge on types of human errors and influencing factors (2.7 vs. 2.6, *p* = 0.024; 3.2 vs. 3.0, *p* = 0.038) while male students showed greater confidence in speaking up (3.5 vs. 3.2, *p* < 0.001) and discussing their own errors (3.5 vs. 3.4, *p* = 0.04), with both expressing equally strong interest in patient safety education.

Students perceive the financial burden on the healthcare system as significantly high due to errors and patient harm (mean 8.1/10) and recognize that antimicrobial resistance is an issue of patient safety. Regarding both issues, students in the clinical years had significantly higher ratings compared with their colleagues from the preclinical years (mean 8.5/10 vs. 7.7/10 and 8.7/10 vs. 7.7/10, respectively, < 0.001). Furthermore, preclinical students evaluated their current education on healthcare quality and patient safety higher (mean 6.6/10 vs. 4.1/10, *p* = 0.004). Almost half of the medical students perceived experiencing an error either as patients (53.3%, *n* = 340) or witnessing one during their clinical training (48%, *n* = 306). However, this experience did not appear to affect their interest or awareness regarding patient safety, as reflected in their overall questionnaire scores (3.13 vs. 3.18, *p* = 0.06) and their expressed desire for future training (92.8% vs. 92.7%, *p* = 0.98).

*Students perceive the financial burden on the healthcare system as significantly high due to errors and patient harm (mean 8.1/10) and recognize that antimicrobial resistance is an issue of patient safety*.

### 3.3 Error Experience During Clinical Training

Among the students who perceived witnessing an error during their undergraduate studies, 81 (26.5%) provided detailed descriptions. Thematic content analysis revealed five primary error types: (a) noncompliance with infection prevention protocols, (b) medication errors, (c) errors in therapeutic management, (d) diagnostic errors and (e) patient identification errors. Most students described errors (47.5%) that pertained to breaches of hygiene protocols, particularly in hand hygiene, the use of protective equipment and the adequate tool sterilization. Illustrative quotes of students' responses are presented along with the main themes in Table 3.

**TABLE 3:** Main themes on types of errors described by undergraduate medical students, with illustrative quotes.

Main themes	Quotes
Noncompliance with infection prevention protocols	‘Placement of urinary catheter without the use of sterile gloves’ (male, 30 years old, 3rd year of study) ‘Total lack of disinfection of equipment, e.g., sphygmomanometer, oximeter, stethoscope are being used from one patient to another.’ (male, 3rd year of study)
Medication errors	‘Prescription of antibiotic without prior culture’ (female, 4th year of study)
Errors in therapeutic management	‘Colon perforation during colonoscopy’ (female, 3rd year of study)
Diagnostic errors	‘They gave an incorrect (serious) diagnosis which worried the patient’ (male, 3rd year of study)
Patient identification errors	‘A non‐diabetic patient was almost administered insulin as they mixed up the beds in the hospital’ (female, 3rd year of study)

### 3.4 Patient Safety Improvement Through Digitalization in Healthcare

A total of 239 students responded (37.5%) to the relevant open‐ended question, with their answers being categorized into seven main themes: (1) Electronic recording systems for patient data; (2) Facilitating error recording and utilization for quality improvement and patient safety; (3) Telemedicine and accessibility; (4) Internet use to support multidisciplinary collaboration and dissemination of guidelines and evidence‐based information; (5) Improving the accuracy of diagnoses, medication management and therapeutic planning; (6) Enhancing organization, accelerating healthcare processes and reducing bureaucracy; and (7) Artificial intelligence. Although the vast majority highlighted the advantages of electronic patient files, which provide comprehensive and accessible patient histories, facilitate integrated care, reduce errors from missing information, and contribute to transparency and legal documentation of medical interventions, a few respondents (8, 3.3%) expressed limited awareness of how digitalization could enhance patient safety. Illustrative quotes on each theme are presented in Table 4.

**TABLE 4:** Main themes on the contribution of digitalization in healthcare on enhancing patient safety through medical students' perspective and illustrative quotes.

Themes	Quotes
Electronic recording systems for patient data	‘I consider the updated electronic health record of the patient a significant step towards improving patient safety, so that all available health information of patients is accessible at any healthcare institution they may visit.’ (female, 5th year of study) ‘Direct access for the treating physician to a complete and detailed medical history and tests' results. That leads to safer care, especially for elderly patients who may not carry all necessary documents or may omit details from their personal history.’ (female, 5th year of study)
Facilitating error recording and utilization for quality improvement and patient safety	‘Electronic reporting of errors, perhaps anonymously, would help in quality improvement studies in hospitals and to better train medical staff’ (male, 3rd year of study) ‘Immediate recording of errors will become easier, data can be collected rapidly and in bulk for evaluation and interventions when common errors are identified.’ (male, 5th year of study)
Telemedicine and accessibility	‘Avoiding contact with nosocomial microbial strains’ (female, 3rd year of study) ‘Possibility of telemedicine and remote communication with the physician.’ (female, 2nd year of study)
Internet use to support multidisciplinary collaboration and dissemination of guidelines and evidence‐based information	‘Easy access to guidelines and protocols’ (male, 3rd year of study) ‘… I also think that Internet facilitates interprofessional collaboration, which can enhance patient safety.’ (male, 3rd year of study)
Improving the accuracy of diagnoses, medication management, and therapeutic planning	‘There could be safety checkpoints in the prescription of certain medications, such as antibiotics’ (female, 4th year of study) ‘Technology‐assisted monitoring allows for accurate diagnosis and treatment, as well as faster response in case of errors.’ (female, 1st year of study)
Enhancing organization, accelerating healthcare processes and reducing bureaucracy	‘Less bureaucracy and thus greater focus on the patient as an individual. This allows for more attention to the patient and helps reduce avoidable problems’ (female, 1st year of study) ‘Routine procedures can be carried out more easily and quickly, allowing more time for complex and challenging tasks.’ (female, 3rd year of study)
Artificial intelligence (AI)	‘Ability to apply AI to complex medical cases’ (male, 5th year of study). ‘Perhaps artificial intelligence could play a key role in detecting errors.’ (male, 3rd year of study)

### 3.5 Expectations on Patient Safety Training Within the Undergraduate Medical Curriculum

Most students (92.8%, *n* = 592) advocated for introducing patient safety concepts into the undergraduate curriculum. They favoured practical guidance on handling errors, speaking up and the error reporting process. Preferred teaching methods included a combination of didactic lectures and experiential learning through real‐life case studies. They also underlined the importance of integrating patient safety principles into the teaching of all clinical courses while also watching their professors acting as role models. Illustrative quotes per main theme are presented in Table 5.

**TABLE 5:** Main themes regarding students' expectations regarding training on PS within the undergraduate medical curriculum with illustrative quotes.

Main themes	Quotes
Course content	‘Focused training on acknowledging and addressing health‐care related errors’ (female, 5th year of study) ‘It would be beneficial to learn how to report an error in healthcare.’ (female, 4th year of study)
Teaching methods	‘Training should be based on real‐life scenarios’ (male, 3rd year of study) ‘Use of simulation tools to practice clinical skills initially in a controlled environment.’ (female, 5th year of study)
Training form	‘I believe that patient safety training should be included in all clinical and related courses, occurring each year of study, allowing for simultaneous exposure to patient safety issues alongside clinical education’ (male, 4th year of study) ‘Certain courses related to communication and error prevention should be offered as mandatory workshops.’ (male, 2nd year of study) ‘Continuous repetition and emphasis, throughout all clinical courses, of the fundamental concepts and practices regarding healthcare quality and patient safety is required.’ (male, 5th year of study)
Teaching professors as role models	‘Aside from the theoretical part of teaching, it is imperative that the medical and teaching staff adhere to everything they teach in the classroom within the clinics and laboratories, as they also serve as role models for the students’ (male, 3rd year of study) ‘Professors should apply in practice what we are taught, thus serving as positive role models. Prolonged exposure of students to incorrect care practices may lead them to commit the same errors they were taught to avoid.’ (male, 4th year of study)

*They also underlined the importance of integrating patient safety principles into the teaching of all clinical courses while also watching their professors acting as role models*.

## 4 Discussion

This survey provided insight into undergraduate medical students' patient safety knowledge, perceptions and error experiences, as well as their perspectives on improving it through training and digitalization. Findings indicate significant knowledge deficits in human error types, contributing factors, risk management, speaking up and error reporting. Students expressed dissatisfaction with the current healthcare system's safety but anticipated that their future workplaces will align with safety culture principles. At the same time, they believe that digital tools could enhance patient safety and organizational processes through electronic data and error recording systems, telemedicine and artificial intelligence. Nearly half had witnessed an error. However, attitudes towards safety culture were encouraging. Students recognized the importance of cultivating relevant skills throughout the curriculum, highlighting the necessity of appropriate educational interventions underlining the importance of the hidden curriculum.

*Digital tools could enhance patient safety and organizational processes through electronic data and error recording systems, telemedicine and artificial intelligence*.

Although students consider the provision of quality healthcare services to be an integral part of their role as future physicians, they report feeling inadequately prepared to address patient safety. These findings can be attributed to the absence of structured, mandatory patient safety modules within undergraduate medical curricula in Greece, indicating that the diffuse and unstructured incorporation of relevant content across preclinical and clinical courses is not adequate. Consistent educational deficits have been documented across diverse contexts. In Ethiopia, 60% of medical and health sciences' students self‐assessed their relevant knowledge as ‘fair’ or ‘poor’ [14], whereas in Pakistan, mean knowledge scores reached only 13/20 [15]. Almost 57% of Tunisian students felt adequately prepared to prevent medical errors [3], and 62% of students in Jordan argued for a good understanding of safety principals because of undergraduate training [5].

*Although students consider the provision of quality healthcare services to be an integral part of their role as future physicians, they report feeling inadequately prepared to address patient safety*.

Students from all years of studies indicated limited familiarity with error reporting procedures, while clinical‐year students felt greater uncertainty about preventing patient harm. In line with these findings, in South Africa, one‐third of final‐year students self‐rated as novices in explaining medical errors [16], whereas at Wayne State University, students described only moderate readiness for handling future adverse events [17]. In most cases, the lowest self‐assessed competencies were noted in speaking up and in the formal error reporting process [18]. However, prior exposure to patient safety education has been shown to foster significantly more favourable perspectives on the incidence and management of medical errors [19].

*Students from all years of studies indicated limited familiarity with error reporting procedures, while clinical‐year students felt greater uncertainty about preventing patient harm*.

Given the recorded levels of relevant knowledge and skills and the fact that only half of our students rated their training in safety and quality above average, the need for curricular reform in undergraduate medical education is evident. Student demand is strong, with 90% arguing for patient safety certification for all medical students and universal agreement on early inclusion of its principles in undergraduate curriculum [20]. This request is in line with the proposals of the National Strategy for Quality of Care and Patient Safety in Greece, that has suggested mandatory patient safety modules [11], as well as with WHO report ‘Taking the pulse of quality of care and patient safety in the WHO European Region’ [21]. Key objectives to be addressed include developing error analysis skills and promoting open discussions about errors [22]. Active, experiential approaches such as interactive cases, problem‐solving and interprofessional simulation are preferred, enhancing critical thinking, teamwork, error recognition and effective management of complex clinical contexts [6, 23]. It is imperative to adopt interprofessional learning, as it fosters essential competencies such as effective teamwork, communication, and shared decision‐making, thereby enhancing collaborative clinical practice and strengthening patient safety and care coordination within healthcare teams [6]. Innovative interventions, ranging from cinemeducation [7], to gamification [24], demonstrate promising directions in fostering engagement and improving safety outcomes.

*Key objectives to be addressed include developing error analysis skills and promoting open discussions about errors*.

Approximately half of participants declared that it would be acceptable to openly admit an error. In previous studies, although one in two expected management to focus on safety issues, significantly fewer anticipated favour for adverse event reporting process [12], with concerns about legal repercussions further discouraging disclosure [17]. Evidence shows that nonpunitive response to errors score lowest in safety culture assessments reflecting blame‐oriented attitudes as reported by nurses in India [25]. Psychological safety is essential for fostering open communication. Without it, hierarchical structures and power differentials in medicine can suppress speaking up, reduce willingness to report errors and ultimately undermine the development of a true safety culture [26].

Regarding error experiences, almost half of participants believed that they had witnessed an error in healthcare delivery during their training. At Jimma University Institute of Health, one‐third had declared witnessing patient harm, with one‐tenth of students admitting to having caused harm during care provision [27]. Technical errors during clinical practices were previously reported, followed by diagnostic, therapeutic, medication, management and communication errors among healthcare professionals. Equally common were the deviations from infection prevention standards, technical and communication errors between doctor and patient [28].

Concerning the contribution of digitalization to promoting patient safety, this is the first study to investigate medical students' perspectives, addressing a critical gap. Students listed among its applications the use of telemedicine and digital tools for supporting diagnostic decisions, remote consultations, therapeutic planning, maintaining electronic health records and facilitating communication among healthcare professionals [29]. Notably, 8 out of 10 students expressed willingness to use digital tools in patient care, with higher readiness among senior years. Reported benefits included improved quality of care, easier access to patient data and increased work satisfaction. However, concerns were raised about stress, delays and financial costs [30]. They reinforced the need for integrating core digital health competencies into the medical curricula to increase digital health literacy and ensure that the future workforce can use digital tools safely, ethically and effectively.

Future research directions should focus on designing and evaluating targeted educational interventions, such as simulation‐based training, safety modules and digital health tools, to address gaps in students' preparedness. Longitudinal and cross‐institutional studies could clarify how perceptions evolve and vary across contexts. Furthermore, qualitative approaches, including focus groups and individual interviews, may provide in‐depth insight into students' perspectives and faculty readiness to teach patient safety concepts.

*Future research directions should focus on designing and evaluating targeted educational interventions, such as simulation‐based training, safety modules and digital health tools, to address gaps in students' preparedness*.

## 5 Strengths and Limitations

This is the first mix‐methods survey that explores patient safety ‘through medical students' eyes’ in Greece and one of the few internationally, after the implementation of the ‘Global Patient Safety Action Plan 2021–2030’ and the COVID‐19 pandemic. Among its strengths is the participation of students from all years of undergraduate studies. Additionally, to the best of our knowledge, medical students' perspectives on the role of digital tools in ensuring patient safety have not been previously studied worldwide.

Certain limitations should be considered when interpreting our results. First, our study was limited to students from one medical school. Even though Aristotle University of Thessaloniki is the largest university in Greece that welcomes students from all over the country after national exams, the findings may not be fully generalizable to other settings, highlighting the need for future multicentre studies to provide a more comprehensive picture. Second, our response rate, although acceptable for online surveys, may have introduced selection bias. In an effort to increase participation rates in future research, providing small incentives for participants and sending personalized informative emails to faculty members regarding the study scope could be tested. Additionally, the limited contribution of sixth‐year students may have led to underrepresentation of perspectives shaped by more extensive clinical exposure, despite most responses being consistent with those of earlier clinical years. Reduced involvement was attributed to their increased workload and their limited presence on campus due to clinical responsibilities in numerous hospital settings. Finally, the open‐ended questions used, despite offering valuable insight from a large number of participants, were inevitably brief, lacking the opportunity to elaborate, clarify, or provide contextual details that could have been achieved through interviews or focus groups. The latter approaches also facilitate exploration of complex or sensitive topics that could be targeted in future surveys.

## 6 Conclusions

Despite positive perceptions and high expectations for safety culture, medical students feel unequipped to manage safety issues and request more knowledge and skills in patient safety. Considering students' needs, introducing patient safety concepts into the undergraduate curriculum, learning through real‐life case studies, as well as integrating patient safety principles into the teaching of all clinical courses should be pursued. Professors acting as role models in handling safety issues could contribute significantly to students' training. Therefore, tailored educational reforms should optimally prepare future physicians to become key players in improving quality of care and patient safety, leveraging the opportunities offered by healthcare digital transformation.

*Tailored educational reforms should optimally prepare future physicians to become key players in improving quality of care and patient safety, leveraging the opportunities offered by healthcare digital transformation*.

## Author Contributions

**Anna Pappa:** conceptualization, investigation, data curation, formal analysis, visualization, project administration, writing – original draft, writing – review and editing. **Athanasios Xymitidis:** investigation, data curation, formal analysis, visualization, writing – original draft. **Thomas Strymponis:** investigation, data curation, formal analysis, visualization, writing – original draft. **Paraskevi Panagopoulou:** methodology, resources, validation, writing – review and editing. **Válter R. Fonseca:** conceptualization, methodology, validation, writing – review and editing. **Henrique Martins:** conceptualization, methodology, validation, writing – review and editing. **João Breda:** methodology, validation, writing – review and editing. **Zoi Tsimtsiou:** conceptualization, methodology, resources, supervision, writing – review and editing.

## Funding

The authors have nothing to report.

## Disclosure

The authors affiliated with the World Health Organization (WHO) are alone responsible for the views expressed in this publication, and they do not necessarily represent the decisions or policies of the WHO.

## Ethics Statement

Ethical approval was obtained from the institutional Bioethics Committee.

## Conflicts of Interest

The authors declare no conflicts of interest.

## Supporting information


**Data S1:** Supporting Information

## Data Availability

The data that support the findings of this study are available on request from the corresponding author. The data are not publicly available due to privacy restrictions.
