# Supplementary material for: Patient Safety ‘Through Undergraduate Medical Students' Eyes’: A Mixed‐Methods Survey
Source: Clin Teach. 2025 Dec 18;23(1):e70332. doi: 10.1111/tct.70332 (PMC12712750; doi:10.1111/tct.70332)

**Supplementary Material**

**Insights Into the Mechanism of Treatment of Ulcerative Colitis With Preprocessed and Postprocessed Dried Ginger by Integrating Serum Pharmacochemistry With Network Pharmacology and Harmacological Validation**

Siming Han, Xiaotong Yang, Jie Du, Zeyu Hou, Wei Wei, Jiahao Li, Xin Yong, Huijun Xu*

Department of Pharmaceutical Analysis, School of Pharmacy, Hebei Medical University, Shijiazhuang, 050017, P. R. China

Correspondence: * Prof. Huijun Xu, Department of Pharmaceutical Analysis, School of Pharmacy, Hebei Medical University, Shijiazhuang 050017, P.R. China

E-mail: xuhuijun36@163.com

Fax: +86-311-86266419

**Content**

**1 Methods**

- 1. **Instruments and Reagents**

**1.2 Animals and groups**

**1.3 UHPLC-Q-TOF-MS analysis conditions**

**1.4 Statistical data processing and analysis**

**Table S1** Mice DAI score

**Table S2** Active ingredients of DG and PG

**Table S3** Molecular docking binding energy

**Figure S1** Compound-Disease targets venny diagram

**Figure S2** PPI network diagram

**1 Methods**

- 1. **Instruments and Reagents**

Instruments：UHPLC-Triple-TOF-MS including: LC-30 A Ultra High Performance Liquid Chromatograph (Shimadzu, Japan), Triple TOF 5600^+^ Mass Spectrometer (AB Sciex, USA); Analytical Balance BP211D (Beijing Sartorius Instruments Co., Ltd., China); MTN-2800D Nitrogen Blow Meter (Tianjin Otesaense Instrument Co., Ltd., China); TOL-20M Table-top High-Speed Freezing Centrifuge (Hunan Xiangyi Laboratory Instrument Development Co., Ltd., China).

Reagents: acetonitrile (chromatographic grade, TEDIA, USA); formic acid and methanol (chromatographic grade, Thermo Fisher Scientific); deionized Water (Hangzhou Wahaha Co., Ltd., China), and all other reagents were analytically pure. DG and PG (Anhui Daoyuantang Traditional Chinese Medicine Drinking Tablets Co.)

- 1. **Animals and groups**

The rats were randomly divided into three groups: control, DG, and PG groups. They were maintained under a 12-hour light-dark cycle with ad libitum access to food and water. After one week acclimatization period, the DG and PG groups were administered orally with DG or PG at a dose of 14 g/kg via gavage for three consecutive days at the same time. The control group was fed normally. On the third day, blood samples were collected from the inner canthus vein at 0.083, 0.17, 0.5, 1, 1.5, 2, 4 and 8 h after the last administration. 0.3 ml of blood was collected each time and were left standing at 4 ℃ for 1 hour and then centrifuged at 3000 rpm at 4 ℃ for 10 min to separate the serum. Serum samples from the same mice were pooled and stored at -80 ℃ for further analysis.

- 1. **UHPLC-Q-TOF-MS analysis conditions**

Chromatographic condition: Separation of the samples was achieved using an Acquity UHPLC HSS T3 column (100 × 2.1 mm, 1.8 μm). The mobile phase consisted of 0.1% formic acid in water (A) and acetonitrile (B). The column temperature was maintained at 40 ℃, and the flow rate was set at 0.3 mL/min. The gradient elution program was as follows: 0–2 min, 10–20% B; 2–15 min, 20–85% B; 15–16 min, 85–100% B; 16–19 min, 100% B; 19–20 min, 100–10% B; 20–21 min, 10% B.

The system was pre-equilibrated for 5 min prior to injection. The injection volume was 5 μL, and the sample chamber temperature was maintained at 4 ℃. The mass spectrometer was controlled and data were acquired using Analyst TF software, while PeakView 2.0 software was used for spectral interpretation and compound identification.

Mass Spectrometric Conditions: Detection was performed using an ESI source in positive ion mode with full scan mode. Real-time calibration was conducted using calibration solutions during the experiment.

The parameters for IDA mode were set as follows: ion source temperature, 550 ℃; nebulizer gas (Gas 1), 55 psi; auxiliary gas (Gas 2), 55 psi; curtain gas, 35 psi; ion spray voltage (ISV), 5500 V; declustering potential (DP), 60 V; collision energy (CE), 15 eV; collision energy spread (CES), 15 eV. The mass range for primary MS scanning was 100–1000 m/z, and for secondary MS scanning, it was 50–800 m/z. Dynamic background subtraction (DBS) was enabled.

- 1. **Statistical data processing and analysis**

By searching the China National Knowledge Infrastructure (CNKI)(https://www.cnki.net/) and PubMed(https://pubmed.ncbi.nlm.nih.gov/), the compound database for DG and PG was established. The self-built database was imported into Peak View, where the elemental composition error and the secondary fragment ion error were respectively set to 5 ppm and 10 ppm. Compound identification was performed using the Master View and IDA functions.

The raw data were imported into Progenesis QI for data preprocessing, including deconvolution and peak alignment. The processed dataset was subsequently analyzed using SIMCA to perform multivariate statistical analyses. Unsupervised principal component analysis (PCA) and supervised orthogonal partial least squares discriminant analysis (OPLS-DA) were performed. The OPLS-DA model was used to maximize intergroup separation, and variable importance in projection (VIP) was employed to rank the contributions of all variables to intergroup differences, thereby identifying compound specificity. Statistical analysis was conducted using MetaboAnalyst 5.0 (<https://www.metaboanalyst.ca/>) to perform independent t-tests to evaluate significant changes in variables. Compounds were considered significantly different between DG and PG when they simultaneously met the criteria of VIP > 1 and P < 0.05.

**Table S1** Mice DAI score

| **Score** | **Percentage weight loss** | **Stool consistency** | **Fecal occult blood or bloody stool** |
| --- | --- | --- | --- |
| 0 | 0 | Normal | Normal |
| 1 | 1-5% | Soft stool | Fecal occult blood |
| 2 | 5-10% | Loose stool | Fecal occult blood |
| 3 | 10-20% | Loose stools | Naked bloody stool |
| 4 | >20% | Diarrhea | Naked bloody stool |

**Table S2** Active ingredients of DG and PG

| **ID** | **Compound name** | **Number of targets** | **Source** |
| --- | --- | --- | --- |
| ZZ1 | Zingerone | 77 | Gingerols |
| ZZ2 | 2-Gingerol | 14 | Gingerols |
| ZZ3 | 4-Shogaol | 100 | Gingerols |
| ZZ4 | 6-Shogaol | 100 | Gingerols |
| ZZ5 | 6-Paradol | 40 | Gingerols |
| ZZ6 | 6-Dehydrogingerdione | 70 | Gingerols |
| ZZ7 | 6-Gingedione | 49 | Gingerols |
| ZZ8 | 6-Gingerol | 88 | Gingerols |
| ZZ9 | 6-Gingerdiol | 100 | Gingerols |
| ZZ10 | 8-Shogaol | 100 | Gingerols |
| ZZ11 | 1-Dehydro-8-gingerdione | 16 | Gingerols |
| ZZ12 | 8-Gingerdione | 77 | Gingerols |
| ZZ13 | 8-Gingerol | 98 | Gingerols |
| ZZ14 | Gingerenone C | 100 | Gingerols |
| ZZ15 | 10-Shogaol | 66 | Gingerols |
| ZZ16 | 1-Dehydro-10-gingerdione | 76 | Gingerols |
| ZZ17 | 10-Gingerdione | 73 | Gingerols |
| ZZ18 | 10-Gingerol | 100 | Gingerols |
| ZZ19 | Gingerenone A | 100 | Gingerols |
| ZZ20 | 12-Shogaol | 16 | Gingerols |
| ZZ21 | Gingerenone B | 82 | Gingerols |
| ZZ22 | Isogingerenone B | 66 | Gingerols |
| ZZ23 | Methyl-6-gingerol | 16 | Gingerols |
| ZR1 | 4-Gingerol | 79 | Gingerols |
| ZR2 | Methyl-10-gingerol | 102 | Gingerols |
| ZR3 | 12-Gingerol | 15 | Gingerols |
| ZR4 | Acetoxy-10-gingerol | 15 | Gingerols |
| ZR5 | Zingerol | 110 | Gingerols |
| ZR6 | Linoleic acid | 15 | TCMSP |
| ZR7 | Copaene | 15 | TCMSP |
| ZR8 | (+)-1,5-Epoxy-nor-ketoguaia-11-ene | 33 | TCMSP |
| ZR9 | α-Glyceryl linoleate | 1 | TCMSP |
| ZR10 | Ginkgetin | 39 | TCMSP |
| ZR11 | Isoginkgetin | 48 | TCMSP |
| ZR12 | Sexangularetin | 36 | TCMSP |
| ZR13 | Daucosterol | 14 | TCMSP |
| ZR14 | β-sitosterol | 1 | TCMSP |
| ZR15 | Sitosterol | 16 | TCMSP |
| ZR16 | Cedrene | 11 | TCMSP |
| ZR17 | Oleic acid | 32 | TCMSP |
| ZR18 | Dibutyl phthalate(DBP) | 46 | TCMSP |
| ZR19 | β-Citraurin | 15 | TCMSP |
| ZR20 | Methyl palmitate | 37 | TCMSP |
| ZR21 | 2-Nonanone | 68 | BATMAN-TCM |
| ZR22 | 1,8-Cineole | 11 | BATMAN-TCM |
| ZR23 | (Z)-Citral | 46 | BATMAN-TCM |
| ZR24 | Geranyl Acetate | 1 | BATMAN-TCM |
| ZR25 | Hexahydrocurcumin | 9 | BATMAN-TCM |
| ZR26 | Zingiberol | 55 | BATMAN-TCM |
| ZR27 | Nonaldehyde | 5 | BATMAN-TCM |
| ZR28 | Geraniol | 13 | BATMAN-TCM |
| ZR29 | Shyobunone | 10 | BATMAN-TCM |
| ZR30 | Campherenol | 24 | BATMAN-TCM |
| ZR31 | (E)-Citral | 41 | BATMAN-TCM |
| ZR32 | Nereistoxin | 1 | BATMAN-TCM |
| ZR33 | 2-Heptanol | 100 | BATMAN-TCM |
| ZR34 | Linalool | 20 | BATMAN-TCM |
| ZR35 | 2-Nonanol | 71 | BATMAN-TCM |
| ZR36 | 2,6-Nonamethylene Pyridine | 14 | BATMAN-TCM |
| ZR37 | Pyrethrin II | 100 | Gingerols |
| ZR38 | α-Cubebene | 12 | Gingerols |
| ZR39 | Methyl Isocyanate(MIC) | 94 | Gingerols |
| ZR40 | Methyl-8-gingerol | 100 | Gingerols |
| ZR41 | Sitogluside | 16 | Gingerols |
| ZR42 | 12,13-Di-acetoxyl-1,4,6,11-eudesmanetetol | 14 | Gingerols |
| ZR43 | Gingerglycolipid B | 15 | Gingerols |
| ZR44 | 1-Monolinolein | 16 | Gingerols |
| ZRP1 | Diacetoxy-6-gingerdiol | 100 | Gingerols |
| ZRP2 | Methyl diacetoxy-6-gingerdiol | 105 | Gingerols |
| ZRP3 | Gingerdione | 113 | Gingerols |
| ZRP4 | slogal | 19 | Gingerols |
| ZRP5 | Oxyphenbutazone | 100 | Gingerols |
| ZRP6 | Zingiberone | 58 | Gingerols |
| ZRP7 | L-Pipecolicacid | 91 | Gingerols |
| ZRP8 | 1-Dehydro-6-gingerdione | 100 | Gingerols |

**Table S3** Molecular docking binding energy

| **Compound** | **Binding energy (kJ/mol)** | | | | |
| --- | --- | --- | --- | --- | --- |
|  | IL6 | TNF | AKT1 | STAT3 | ALB |
| 8-Gingerol | -5.8280 | -5.3974 | -5.7620 | -5.3367 | -6.5511 |
| 8-Shogaol | -5.3307 | -5.7996 | -5.7231 | -5.3396 | -5.8195 |
| Gingerdione | -5.7166 | -5.3782 | -5.1333 | -5.4218 | -6.1712 |
| Gingerenone A | -6.5872 | -6.2305 | -5.3602 | -6.1332 | -5.9811 |
| Oxyphenbutazone | -5.4822 | -5.0666 | -5.4662 | -5.0188 | -5.7746 |

**Figure S1** Compound-Disease targets venny diagram


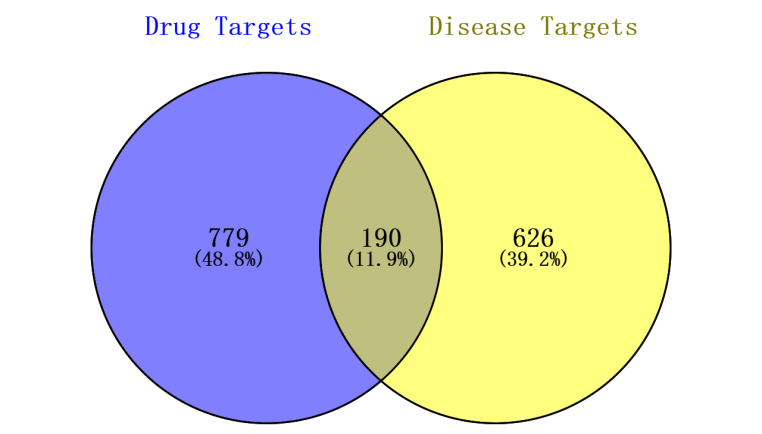


**Figure S2** PPI network diagram


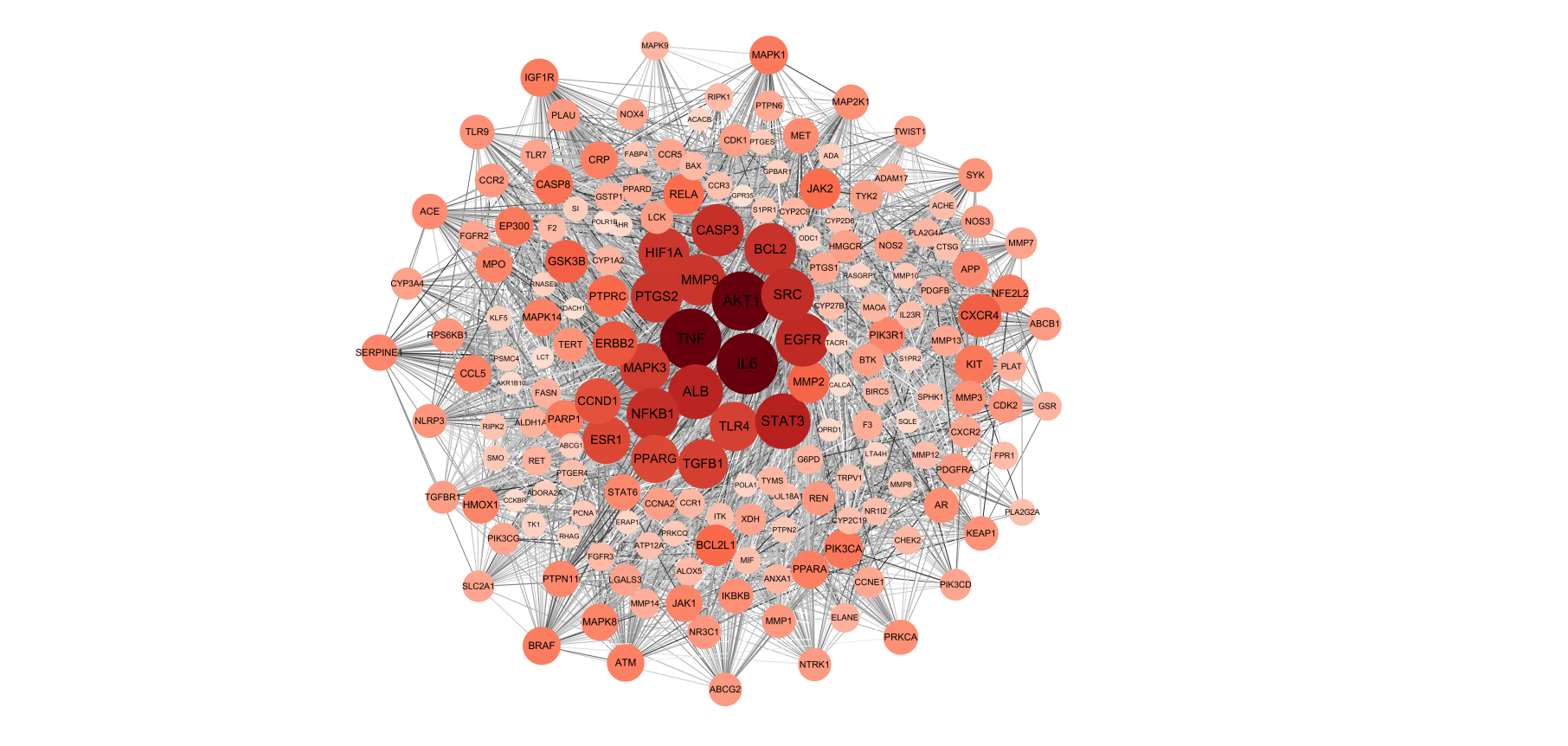

Supplement: Supplementary file 1 — Data S1: Supporting Information [file TCT-23-e70332-s001.docx]
